# Transcutaneous bilirubin-based screening reduces the need for blood exchange transfusion in Myanmar newborns: A single-center, retrospective study

**DOI:** 10.3389/fped.2022.947066

**Published:** 2022-09-06

**Authors:** Hiromi Suzuki, Saneyuki Yasuda, Yinmon Htun, Nant San San Aye, Hnin Oo, Thet Paing Oo, Zaw Lin Htut, Kosuke Koyano, Shinji Nakamura, Takashi Kusaka

**Affiliations:** ^1^Department of Hygiene, Faculty of Medicine, Kagawa University, Kagawa, Japan; ^2^Post Graduate Clinical Education Center, Kagawa University Hospital, Kagawa, Japan; ^3^Department of Pediatrics, Faculty of Medicine, Kagawa University, Kagawa, Japan; ^4^Neonatal Intensive Care Unit, Central Women’s Hospital, Yangon, Myanmar; ^5^Poole Hospital, University Hospitals Dorset NHS Foundation Trust, Poole, United Kingdom

**Keywords:** blood exchange therapy, neonatal jaundice, infants, transcutaneous bilirubin, phototherapy, nomogram

## Abstract

**Background:**

Neonatal hyperbilirubinemia is a significant health problem in Myanmar. We introduced transcutaneous bilirubin (TcB) measurements in 2017 and developed an hour-specific TcB nomogram for early detection and treatment of hyperbilirubinemia in Myanmar neonates. This study aimed to evaluate whether our screening method for hyperbilirubinemia decreased the requirement of blood exchange therapy (ET).

**Methods:**

This retrospective cohort study was conducted at the Central Women’s Hospital, Yangon. Two groups were included as follows: group 1 (control group; comprising infants born in 2016 and screened on the basis of Kramer’s rule), and group 2 (intervention group; comprising infants born in 2019 and screened by TcB measurement using a nomogram). The number of ETs was analyzed based on causes of hyperbilirubinemia and number of days after birth.

**Results:**

Groups 1 and 2 comprised 12,968 and 10,090 infants, respectively. Forty-six and two infants in Groups 1 and 2, respectively, required an ET. The odds ratio for ET was 18.0 (Group 1 to Group 2; 95% confidence interval [CI]: 4.8–67.1; *p* = 0.000). Serum bilirubin values at the time ET was administered were significantly higher in Group 1 than those in Group 2 (median: 23.0 and 16.8, respectively).

**Conclusion:**

The management of hyperbilirubinemia using our screening method (TcB Nomogram) can effectively reduce the need for ET in neonates in Myanmar.

## Introduction

Neonatal hyperbilirubinemia presents a significant health burden in developing countries, such as Myanmar (1, 2). It must be detected and treated early to prevent irreversible bilirubin encephalopathy, permanent neurological deficits, or death of the neonates (3). Phototherapy is an effective non-invasive treatment for neonatal hyperbilirubinemia; early detection of hyperbilirubinemia enables the administration of phototherapy at hospitals, even in developing countries. However, late detection may lead to extreme hyperbilirubinemia, which requires invasive treatments [such as blood exchange transfusion (ET)] to prevent bilirubin encephalopathy and death (4). A previous intervention study conducted in Myanmar revealed that intensive phototherapy could significantly reduce ET rates (5). Therefore, early detection and timely treatment with phototherapy are important to avoid tragic outcomes (6).

In Myanmar, the majority of newborns are delivered at home and may not receive adequate medical care at birth, including home visits and physical examinations by doctors. Some parents do not seek hospital checkups unless the infant shows obvious anomalies or neurological abnormalities. Home birth and self-referral to a hospital are significant risk factors for the presentation of acute bilirubin encephalopathy (7). On the other hand, infants born at hospitals are likely to undergo regular medical observation and examination for hyperbilirubinemia based on Kramer’s rule (8), which is highly subjective.

Kramer’s rule identifies hyperbilirubinemia based on the dermal change of color on the head, hands, and feet (8). The Kramer score is indicated as follows: (1) jaundice of head and neck; (2) trunk to the umbilicus; (3) groin, including the upper thighs; (4) knees and elbows to ankle and wrists; (5) feet and hands, including palm and soles (8). We are required to score jaundice under natural light by blanching the skin. This method depends on the doctors’ judgment based on their knowledge and experience, and on whether the total serum bilirubin level has been checked. Once the total serum bilirubin level is confirmed objectively, newborns are treated with phototherapy or ET according to the American Academy of Pediatrics (AAP) guidelines (6). However, a recent study in Indonesia evaluated the Kramer score as an invalid method for identifying infants in need of treatment (9).

In 2017, we introduced transcutaneous bilirubin (TcB) measurement in a hospital in Yangon based on previous reports of its usefulness in developing countries in terms of feasibility (10–12). As previously reported, TcB nomograms vary among different ethnicities (13). Without considering ethnic differences, we might misjudge the appropriate timing of treating hyperbilirubinemia. For instance, TcB measurements tend to be overestimated in Myanmar newborns with a dark skin tone and underestimated in those with a light skin tone (14). If these Myanmar newborns are assessed using a nomogram that is intended for a different ethnicity with a lighter skin tone, their TcB values will most likely be above the treatment line; thus, they will be over-treated. Therefore, it is necessary to develop a nomogram specific for each target population. Indeed, with the approval of the Ethics and Review Committee of Myanmar, we successfully developed an hour-specific TcB nomogram to achieve early detection for Myanmar newborns in 2017 (15).

Pediatricians and postgraduate students working at a neonatal unit at the hospital were offered training sessions on the use of TcB measurements and the nomogram developed by us. They have started to include hyperbilirubinemia screening with TcB measurement in routine work at the postnatal wards since 2018. Moreover, because this screening method is feasible, it has been widely used by medical professionals. In this study, we evaluated the effectiveness of this new screening method in reducing the number of ET cases.

## Materials and methods

**FIGURE 1:**
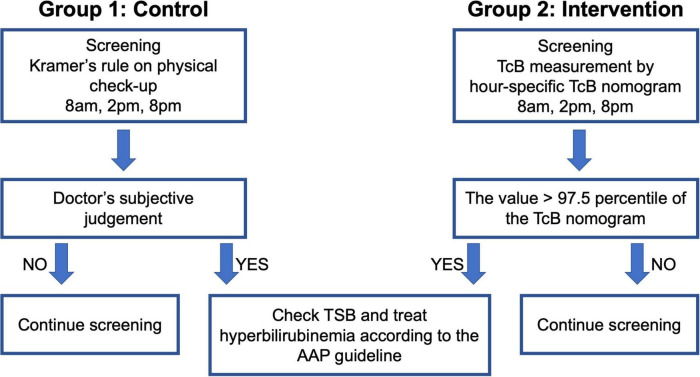
Algorithm of the control and intervention groups for the treatment of hyperbilirubinemia.

This single-center, retrospective cohort study included neonates born at the Central Women’s Hospital Yangon, Myanmar after 35 weeks of gestation with a birthweight > 2,000 g. These infants were divided into the following two groups (Figure 1): Group 1 (control group; infants born between January and December 2016 who were screened by Kramer’s rule), and Group 2 (intervention group; infants born between January and December 2019 who were screened by TcB measurement using our nomogram). Baseline data were retrieved from the annual reports of the Central Women’s Hospital Yangon for the years 2016 (16) and 2019 (17). Data on the date of birth, gestational age, sex, birth weight, mode of delivery, cause of hyperbilirubinemia, and days after birth, were recorded when ET was performed.

**FIGURE 2:**
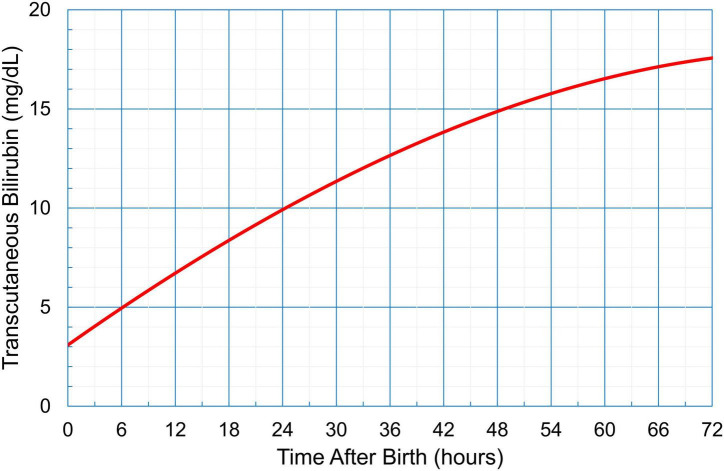
An hour-specific transcutaneous bilirubin nomogram for neonates in Myanmar (gestational age ≥ 35 weeks, birthweight > 2,000 g). This nomogram card was distributed to medical professionals for detecting neonatal hyperbilirubinemia.

In Group 1, the management of hyperbilirubinemia was based on Kramer’s rule; the neonates were checked three times a day (8 AM, 2 PM, and 8 PM, which were the timings for postnatal checkups) from day 0 to day 3 after birth, and they were treated according to the AAP guidelines using the total serum bilirubin (TSB) level determined during blood sampling. In Group 2, TcB was measured by trained doctors using the JM-105 (Konica Minolta, Tokyo, Japan); the measurements were performed three times a day (8 AM, 2 PM, and 8 PM, which were the timings for postnatal checkups) from day 0 to day 3 after birth. The mid-sternum point on the chest of the neonates was chosen as the site of measurement, and the median value of the three measurements was plotted on the hour-specific TcB nomogram (Figure 2). If the value was higher than the 97.5th percentile of the nomogram, a physician collected a blood sample and followed the AAP guidelines for treatment (Figure 1). Treatment included phototherapy or ET.

Opt-out was implemented given the retrospective cohort study design, which precluded the acquisition of informed consent prior to the study. A notice on the research and opt-out policies was put up on the wall at the neonatal unit, for the guardians of the sample infants. This study was approved by the Ethical Committee Registry of the Faculty of Medicine Kagawa University (2021-225) and conducted in accordance with the principles described in the Declaration of Helsinki.

### Statistical analysis

Categorical variables are presented as absolute numbers with percentages. Continuous variables are presented as medians with minimum and maximum values. The odds ratio of the number of ETs in Group 2 against the number of ETs in Group 1 was calculated using Microsoft Excel (Microsoft, Redmond, WA, United States).

## Results

**FIGURE 3:**
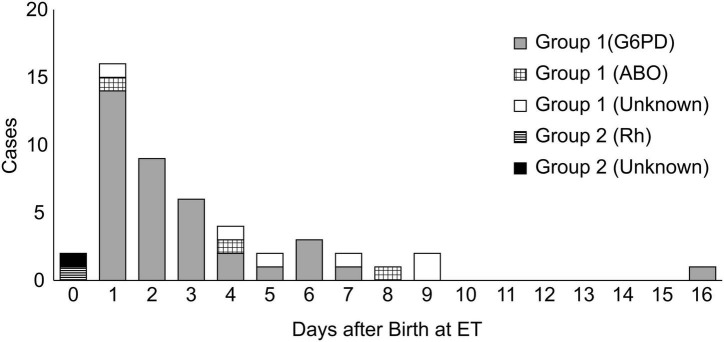
The number of cases that required ET according to the days after birth and causes of hyperbilirubinemia.

We initially included 15,254 neonates, of which 12,968 live-born neonates were categorized into Group 1 (2016), and 12,371 neonates, of which 10,090 were categorized into Group 2 (2019). Forty-six infants were treated with ET in Group 1, while only two infants were treated with ET in Group 2. The odds ratio for ET was 18.0 (Group 1 to Group 2; 95% confidence interval [CI]: 4.8–67.1; *p* = 0.000; Table 1). In Group 1, 46 cases received ET, of which 37 cases (80.4%) had G6PD deficiency as the cause of hyperbilirubinemia. In addition to G6PD deficiency, 11 out of 37 cases (29.7%) had an ABO incompatibility and one case had an Rh incompatibility. The etiology of six cases was unknown. Figure 3 labels all 37 cases as “G6PD deficiency” requiring ET, regardless of the presence of an ABO or Rh incompatibility as the additional cause of hyperbilirubinemia. ET was performed between days 1 and 16 after birth, and day 2 was the median for ET administration in Group 1 (Table 2 and Figure 3). Of the two ET cases in Group 2, one baby was born to an Rh-negative mother and the cord blood sample revealed a positive direct Coombs test and anemia. The second baby exhibited hemolytic jaundice of unknown etiology, with a family history of severe anemia and blood transfusion at birth in his elder sibling. Both babies in Group 2 underwent ET on day 0 (Table 2 and Figure 3), and no baby with G6PD deficiency received ET. Serum bilirubin values at the time ET was administered were significantly higher in Group 1 than those in Group 2 (median: 23.0 and 16.8, respectively) (Table 2).

**TABLE 1:** Characteristics of the Group 1 and Group 2 neonates born at the Central Women’s Hospital Yangon, Myanmar.

Characteristics	*n* (%)	
	Group 1	Group 2
Study population	12,968	10,090
Hospitalized neonates	2,932 (22.6)*	2,049 (20.3)*
Sex (Male)	1,622 (55.3) **	1,119 (54.6) **
Delivery (VD: CS)	1,339 (45.7) : 1,593 (54.3)	901 (44.0) : 1,148 (56.0)
Hyperbilirubinemia	2,067 (70.5) **	1,169 (57.1) **
Phototherapy	2,020 (68.9) **	1,133 (55.3) **
ET	46 (1.6) **	2 (0.1) **

VD, normal spontaneous vaginal delivery; CS, cesarean section; ET, blood exchange transfusion cases which met the inclusion criteria for this study. *Divided by the number of study population. **Divided by the number of hospitalized neonates.

**TABLE 2:** Patient characteristics and causes of hyperbilirubinemia in neonatal cases requiring ET in Groups 1 and 2.

Characteristics	*n* (%), median (min-max)	
	Group 1 (*n* = 46)	Group 2 (*n* = 2)
Sex (Male)	28 (60.9) : 18 (39.1)	2 (100) : 0 (0)
Delivery (VD: CS: V: F)	25 (54.3) : 19 (41.3) : 1 (2.2) : 1 (2.2)	0 : 1 (50) : 1 (50) : 0
BW	3,000 (2,100–4,400)	2,900 (2,500–3,300)
G6PD deficiency	37 (80.4)	0 (0)
Day at ET	2 (1–16)	0 (0–0)
SB values at ET	23.0 (17.3–33.0)	16.8 (6–13.8)

VD, normal spontaneous vaginal delivery; CS, cesarean section; V, vacuum; F, forceps; Day at ET, day after birth which blood exchange transfusion was administered; SB values at ET, serum bilirubin values at day ET was administered.

## Discussion

Our study showed that the management of hyperbilirubinemia using TcB measurement and the nomogram significantly reduced the number of ETs, especially in patients with G6PD deficiency. This study is the first to demonstrate that the early detection of hyperbilirubinemia with TcB measurement is feasible in Myanmar and that the nomogram is very effective in managing neonatal hyperbilirubinemia. Indeed, this new screening method can be easily introduced in all neonatal units of hospitals in the country.

A major concern related to ET in Myanmar is the shortage of human resources. ET requires two doctors (one experienced and one unexperienced doctor), and two nurses (one to assist and one to run errands). Furthermore, neonatal patients need to be intensively cared for after ET. In addition, extra care will be needed if any serious complications occur, such as thromboembolism, necrotic-enteritis, arrhythmia, metabolic abnormalities, abnormal coagulation abnormalities, and infections (18). Therefore, the risk of consuming the limited human resources is a big challenge, especially given that human resources for health are below the WHO recommended level in Myanmar. Another concern is the limitations related to the screening of transfusion-transmissible infections. The only tests available are serology tests for malaria, hepatitis B, C, and HIV. CMV screening test, on the other hand, cannot be routinely performed. To avoid transfusion-related diseases, some prefer blood donation from family members. However, when the blood is not readily available in the blood bank, searching for the donor can be troublesome. Accordingly, the ET-related costs are very substantial in terms of human resources, medical care, and time. Therefore, if possible, ET should be prevented.

In our study, most patients that underwent ET had G6PD deficiency, which is common in malaria-endemic areas, such as tropical Africa and tropical and subtropical Asia (19). G6PD deficiency has been observed in the Myanmar population (20), with a prevalence rate of 6.1% (21). According to the AAP guidelines adopted in Myanmar, where screening for G6PD is not performed on a routine basis, neonatal assessment including the Beutler fluorescent spot test should be performed when neonates develop hyperbilirubinemia {bilirubin concentrations greater than the 95th percentile [150 μmol/L (8.8mg/dL)]} within the first 24 h of life (6). However, hyperbilirubinemia caused by G6PD deficiency is evident at 1–4 days of age, and its manifestation is similar to that of physiological jaundice (19), which is present in approximately 60% of the term newborns in the first week of life (22). This makes it difficult to suspect neonates with G6PD deficiency on day 0, thereby leaving them uncared for until hyperbilirubinemia is so obvious that they need ET. This fact explains why most neonates in this study were treated with ET on days 1 or 2 of life or latest on day 16 in 2016.

Central Women’s Hospital, this study’s site, had similar results to two previous hospital-based studies, which showed a similar incidence of G6PD deficiency in male infants (9%) (23, 24). Significant hyperbilirubinemia occurred in newborn neonates with G6PD deficiency (62.9%), whereas only 15.4% of severe cases were without G6PD deficiency; the ET rates were 3.2% and 0.2%, respectively. Not all G6PD-deficient patients had severe hyperbilirubinemia requiring ET during their hospital stay (23, 24). Since the incidence of G6PD deficiency is relatively high in this population, routine cord blood screening would be helpful to detect G6PD deficiency in Myanmar. Due to limited resources, however, the screening will not be routinely implemented. While male infants are to be tested for their G6PD status at Central Women’s Hospital if they develop jaundice during their hospital stay, female infants are to be tested only if they need ET.

Thus, while the early detection of G6PD deficiency is difficult, the early detection of hyperbilirubinemia is feasible, particularly by following the screening method described in this study. Once hyperbilirubinemia is detected, irrespective of the cause, its timely and effective management by phototherapy, a non-invasive treatment, is possible. Consequently, early detection reduces the incidence of ET in patients with G6PD deficiency.

In 2019, two infants with hyperbilirubinemia were detected by our screening method and underwent ET on the day of birth. One baby had significant hyperbilirubinemia due to an Rh incompatibility, and the other one was found to have hemolytic anemia at 6 h after birth. Interestingly, no patient with G6PD deficiency needed ET. The results demonstrated that our screening method could successfully detect all cases of significant hyperbilirubinemia and avoid unnecessary ET and its complications by providing early non-invasive interventions.

Our study, which analyzed a screening method involving TcB measurements, identified an effective early detection of neonatal hyperbilirubinemia. With Kramer’s rule, TSB reached between 17.3 to 33.0 mg/dL when ET was administered, but TcB measurements could lead to early detection with TSB rising only between 6 to 13.8 mg/dL. Early detection leads to early treatment, which is a recommended convention as per the AAP guidelines. Indeed, we found that the number of cases requiring ET significantly differed between 2016 and 2019.

There is a high risk for healthcare malfunctioning and/or mismanagement in situations where new medical management protocols are introduced. However, our study showed that after the screening method was introduced, no technical or feasibility challenges were observed at the healthcare and medical centers. This is crucial for developing countries, such as Myanmar. Therefore, we recommend that this screening method be expanded to other hospitals in Yangon, provincial areas, and remote areas where medical resources are scarce. Such improvements in neonatal care may decrease the neonatal morbidity and mortality rates in Myanmar.

There are some limitations in our study that should be acknowledged. First, this was a single-center study. The hospital is a tertiary medical institution run by the government and is located in an urban area. Thus, it might be difficult to generalize the results to the entire country. Second, this was a retrospective study; accordingly, we could not prove causality between the screening method and the early treatment administered through phototherapy. Third, the reasons why some cases of hyperbilirubinemia led to ET are unknown because medical examinations are limited in Myanmar; routine examinations are limited to G6PD screening and ABO/Rh incompatibility, and do not include blood complete picture, bleeding and clotting profiles, cerebral hemorrhage, metabolic diseases, abnormalities on thyroid gland, or scrutiny on infection. Fourth, the contribution of this screening method to the early detection of severe neonatal hyperbilirubinemia 72 h after birth remains unclear, because the TcB nomogram for Myanmar neonates was applicable within 72 h of birth.

In conclusion, the management of hyperbilirubinemia with our screening method using an hour-specific TcB nomogram and TcB measurement is effective in reducing ETs in Myanmar neonates. Our study might contribute to a reduction in the medical burdens in neonatal care. Further research is urgently needed to examine the effectiveness of this screening method in other hospitals in different areas, especially where medical resources are scarce.

## Data availability statement

The original contributions presented in this study are included in the article/Supplementary material, further inquiries can be directed to the corresponding author/s.

## Ethics statement

The studies involving human participants were reviewed and approved by Ethical Committee Registry of the Faculty of Medicine Kagawa University (2021-225). Written informed consent from the participants or their legal guardian/next of kin was not required to participate in this study in accordance with the national legislation and the institutional requirements.

## Author contributions

HS, SY, and YH conceptualized the study. HS, SY, NA, and TK designed the study. HS, SY, YH, TO, and ZH collected and analyzed the data. SY, NA, HO, and TK coordinated and supervised the data collection. HS drafted the initial manuscript. SY, YH, KK, SN, and TK critically reviewed and revised the manuscript. All authors approved the final manuscript as submitted and agreed to be accountable for all aspects of the work.
