# Supplementary material for: Transcutaneous bilirubin-based screening reduces the need for blood exchange transfusion in Myanmar newborns: A single-center, retrospective study
Source: Front Pediatr. 2022 Sep 6;10:947066. doi: 10.3389/fped.2022.947066 (PMC9485474; doi:10.3389/fped.2022.947066)
Supplement: Supplementary file 1 [file Table_1.docx]

**The Supplementary Material**

|  | Sex | GA | BW | MOD | Cause | Day at ET | SB values (mg/dL) |
| --- | --- | --- | --- | --- | --- | --- | --- |
| **Group 1** |  |  |  |  |  |  |  |
| 1 | M | Term | 3,500 | VD | G6PD | 2 | >range |
| 2 | M | Term | 2,800 | VD | G6PD | 2 | 20.9 |
| 3 | M | Term | 4,200 | CS | G6PD | 6 | 28 |
| 4 | F | Term | 3,000 | CS | G6PD | 1 | 21.6 |
| 5 | F | Term | 2,500 | CS | G6PD+ABO | 4 | 25.2 |
| 6 | M | Term | 3,900 | CS | G6PD+ABO | 1 | 21.5 |
| 7 | F | Term | 2,300 | VD | Unknown | 1 | 22.6 |
| 8 | M | Term | 3,400 | VD | Unknown | 9 | missing |
| 9 | M | Term | 2,500 | CS | G6PD | 1 | 22 |
| 10 | M | Term | 4,110 | CS | G6PD+ABO | 1 | 17.3 |
| 11 | F | Term | 2,800 | VD | G6PD | 2 | 27.2 |
| 12 | M | Term | 3,300 | VD | G6PD | 16 | 33.0 |
| 13 | F | Term | 4,400 | CS | G6PD | 2 | 30.9 |
| 14 | M | Term | 3,400 | VD | G6PD | 1 | 21.5 |
| 15 | F | Term | 3,000 | CS | G6PD+ABO | 3 | 24.6 |
| 16 | F | Term | 3,700 | VD | G6PD | 3 | 25.1 |
| 17 | M | Term | 3,000 | CS | G6PD | 1 | 23.5 |
| 18 | M | Term | 2,300 | VD | unknown | 7 | 26.6 |
| 19 | M | Term | 2,600 | CS | G6PD | 7 | 23.5 |
| 20 | M | Term | 2,950 | VD | G6PD+ABO | 1 | 23 |
| 21 | M | Term | 2,350 | VD | G6PD | 6 | 23.9 |
| 22 | F | Term | 2,550 | VD | G6PD+Rh | 3 | 25.8 |
| 23 | M | Term | 2,100 | VD | G6PD | 1 | 27.5 |
| 24 | M | Term | 2,800 | CS | unknown | 5 | 25.6 |
| 25 | F | Term | 3,100 | vacuum | G6PD | 1 | 20.8 |
| 26 | M | Term | 2,970 | CS | ABO | 4 | 20.3 |
| 27 | M | Term | 2,300 | VD | Unknown | 4 | 19 |
| 28 | M | Term | 3,250 | CS | G6PD | 4 | missing |
| 29 | F | Term | 2,750 | VD | G6PD+ABO | 1 | 25 |
| 30 | F | Term | 3,000 | VD | G6PD | 5 | 23.3 |
| 31 | M | Term | 3,650 | VD | ABO | 1 | 21.1 |
| 32 | M | Term | 3,110 | VD | G6PD | 1 | 19.7 |
| 33 | F | Term | 2,500 | VD | G6PD+ABO | 3 | 22 |
| 34 | M | Term | 2,700 | VD | G6PD+ABO | 1 | 19.2 |
| 35 | M | Term | 2,600 | CS | G6PD+ABO | 2 | 22.8 |
| 36 | M | Term | 2,800 | VD | G6PD | 2 | 27 |
| 37 | M | Term | 3,400 | VD | G6PD+ABO | 2 | 20 |
| 38 | M | Term | 2,850 | CS | G6PD | 1 | 19.6 |
| 39 | F | Term | 3,000 | VD | G6PD+ABO | 3 | 25 |
| 40 | F | Term | 3,070 | CS | ABO | 8 | 28.4 |
| 41 | M | Term | 3,300 | CS | G6PD | 2 | 22 |
| 42 | M | Term | 3,500 | VD | G6PD+ABO | 1 | 22.9 |
| 43 | F | Term | 3,000 | VD | G6PD | 2 | 23.2 |
| 44 | F | Term | 3,300 | CS | G6PD | 6 | 23.5 |
| 45 | F | Term | 3,900 | CS | G6PD | 3 | 20.9 |
| 46 | F | 36 | 2,300 | Forcep | Unknown | 9 | 23.0 |
| **Group 2** |  |  |  |  |  |  |  |
| 1 | M | Term | 2,500 | CS | Unknown | 0 | 13.8 |
| 2 | M | Term | 3,300 | Vacuum | Rh | 0 | 6 |

GA: gestational age, BW: Birth weight, MOD: mode of delivery, VD: normal spontaneous vaginal delivery, CS: Cesarean section, Day at ET: day that blood exchange therapy was administered, G6PD glucose-6-phosphate dehydrogenase deficiency, ABO: ABO incompatibility, Rh: Rh incompatibility, ET: blood exchange transfusion, SB values: serum bilirubin values at day ET was administered, >range: the value was too high to measure by the machine.
